# How digital economy shapes older adult care services: evidence from China

**DOI:** 10.3389/fpubh.2025.1610952

**Published:** 2025-07-09

**Authors:** Qingyang Bian, Zihe Kong, Jiangming Kong

**Affiliations:** ^1^School of Public Affairs, Zhejiang University, Hangzhou, China; ^2^Jinhua Hospital of Traditional Chinese Medicine, Zhejiang Chinese Medical University, Jinhua, China

**Keywords:** digital economy, older adult care service, technological innovation, older adult care industry, social support, social governance

## Abstract

**Background:**

In the context of an aging society and the burgeoning development of the silver economy, enhancing older adult care services has emerged as a pivotal concern. The technological advancements and industrial shifts driven by the digital economy have provided a novel solution to this challenge. Besides, social organizations, as institutionalized networks of social relations, are important providers of social support for older adult people.

**Methods:**

Drawing on panel data from 30 provinces in China over the period from 2013 to 2023, this paper empirically investigates the impact mechanism through which the digital economy affects older adult care services.

**Results:**

It is found that the digital economy can enhance the quality of older adult care services, primarily by boosting innovation in older adult care-related corporations and driving the development of the older adult care industry (industrial agglomeration and increase in output value). Meanwhile, the positive impact of the digital economy on older adult care services is further strengthened by the social support provided by social organizations. Additionally, the digital economy can also facilitate the improvement of older adult care services in neighboring provinces to a certain extent through spatial spillover effects.

**Conclusion:**

The findings of this paper offer policy insights and confidence for accurately understanding the relationship between the digital economy and older adult care services, and for effectively enhancing older adult care services through the development of the digital economy and the improvement of social governance.

## 1 Introduction

The issue of population aging is rapidly spreading across the globe. By the late 2070s, the global population aged 65 and above is projected to exceed the number of people under 18 (1), with the pace of aging far surpassing historical rates (2). China, a region with one of the largest older adult populations, saw its population aged 60 and above exceed 300 million by the end of 2024 (3). This rapid, large-scale aging trend—alongside the increasingly diverse and complex needs of older adults, including mental, psychological, caregiving, daily living, recreational, environmental, medical, preventive healthcare, hospice care, continued growth, and reemployment demands (4, 5)—presents both obstacles and prospects for global older adult care services.

On the one hand, in the context of the silver economy, older adult individuals, particularly in economically developed regions, receive pensions and possess higher savings and incomes. This endows them with the economic capacity to purchase necessary older adult care goods and services (6, 7), thereby driving related innovation and entrepreneurship on the supply side (8). On the other hand, the current social burden of older adult care is substantial, encompassing the shortage of older adult care professionals and inadequate social security (9). The supportive social older adult care environment, including social care, age-friendly adaptations, and service integration, has not been able to meet the needs of the aging society well (10, 11). This inadequacy adversely affects the physical and mental health of older adults as well as their ability to contribute meaningfully to society. By achieving breakthroughs in advanced technology, innovatively allocating production factors, and deeply transforming and upgrading industries, the digital economy is demonstrating significant development potential in the field of older adult care services (12, 13). New digitalized older adult care service models, such as smart older adult care communities, the integration of medical care, and time banking, continue to emerge (14–17), bringing substantial potential for improving the quality of older adult care services.

While the role of digital technology has been concerned by existing literature (18, 19), most studies remain confined to case analyses or micro-level observations without conducting macro-level systematic investigations that account for the distinctive characteristics of both the digital economy and older adult care serves. The limited macro-level empirical research on related sectors like healthcare (20) has notably overlooked such essential factors as innovation capacity, the transition of older adult care industry, and social support. The role of the digital economy in promoting scientific and technological innovation as well as industrial transition is unparalleled compared to that of traditional economic forms, which is why this paper pays special attention to it. Furthermore, given the quasi-public service nature of China’s older adult care services, the potential influences of social support or social governance environments cannot be ignored scholarly.

Drawing on panel data from 30 provinces in China over the period from 2013 to 2023, this paper empirically investigates the impact of the digital economy on older adult care services and its underlying mechanisms. Specifically, the impact pathway of the digital economy on older adult care services is identified from two dimensions: innovation in older adult care-related corporations and the development of the older adult care industry (including industrial agglomeration and industrial value-added). The paper also examines the crucial moderating role of social organizational support within the older adult care ecosystem in shaping how the digital economy affects older adult care services. Furthermore, the spatial spillover effect of the digital economy on surrounding older adult care services is explored.

This research offers valuable theoretical insights into the intricate relationship and underlying influence mechanisms among the digital economy, social organizational support, and older adult care services. Firstly, this study demonstrates that the technological advancements fostered by the digital economy and the evolution of the older adult care industry can create positive spillover effects in the broader domain of social services, encompassing older adult care. Second, this paper verifies that the combined development of the digital economy and social organizations can more effectively promote the improvement of older adult care services, and supports the notion that the digital economy and social organizations’ participation in social governance have a synergistic effect on social services. Third, previous research has not fully revealed the positive spatial spillover effects of the digital economy on adjacent older adult care services. This study provides new inspiration and confidence for promoting balanced regional older adult care services in the context of the digital economy.

## 2 Research hypotheses

In line with the dynamic capability theory, the rise of the digital economy expedites the absorption, diffusion, application, and integration of digital technologies within enterprises, thereby bolstering their capacity for innovation (21, 22). Older adult care-related corporations, after integrating digital technologies, have achieved innovations in various technical and product aspects, becoming effective solutions for how to provide socially sustainable care services for an aging population (23), thereby promoting the older people’s quality of life and health management (24, 25).

Concurrently, the digital economy has hastened the transformation of the “tech-economic” paradigm, and facilitated the integration of digital technologies throughout the length of the industrial and value chains (26). The digital economy modifies the conventional operational framework of the older adult care sector, while also injecting fresh impetus and prospects for its transformation (20). By integrating sensors, mobile devices, and communication technologies, the smart older adult care platform enables the older adults to perform real-time medical monitoring at home and enjoy high-quality healthy older adult care services (27). Moreover, the digital economy has altered interaction patterns on both the supply and demand sides, pushing enterprises to break away from the limitations of traditional business models (28). This transformation enables firms to leverage digital technologies to precisely identify older adult consumers’ needs and obtain timely product feedback (29), thereby achieving better alignment between senior demands and corporate product innovation. Such digital-enabled matching mechanisms have significantly expanded both material and recreational consumption among the older adult population (30), ultimately facilitating access to better older adult care products and services.

*Hypothesis 1*: The digital economy positively impacts older adult care services.

*Hypothesis 2*: The digital economy enhances the quality of older adult care services through fostering innovation in older adult care-related corporations and advancing the older adult care industry.

The socio-ecological model (31, 32), social capital theory (33), and social cognitive theory (34) all highlight the crucial role of social factors, especially social support, in meeting needs, addressing problems, and maintaining or altering behaviors. Scholars agree that the connotation of social support is multi-faceted and varied (35). In terms of the scope of support, it includes accessible supportive resources, emotional help, informational help, material and cognitive support or sharing some affairs, interaction, etc. (36–38). In 2011, the WHO recognized social support as a crucial factor in promoting active ageing. Extensive research has also shown that substantial social support is closely correlated with enhanced physical and mental wellbeing in older adults, as well as successful ageing (39–41).

Social organizations, as institutionalized networks of social relations, are important providers of professional and external social support that can correct and compensate for government failures, market failures, and family failures (42–44). On the one hand, social organizations have a more flexible structure, with multiple funding sources, a diverse workforce, and a greater range of structural and functional resilience compared to the public and private sectors (45). On the other hand, it has the concept of non-profit, voluntary, autonomous and maximum benefit at minimum cost (46). These characteristics have advantages in terms of content flexibility, the need for a professional approach, and the provision of meager profits, emotional infusion, day care, counseling, nursing and other older adult care services (47). Previous studies have shown that technologically literate mediators from local communities or voluntary organizations can help to promote social connection, address trust issues, and lower obstacles for older people in accessing and utilizing complex digital information via training and other methods (48). Social support from social organizations can also help older people adapt to telemedicine and better benefit from the digital economy (49, 50). Therefore, it can be reasonably judged that in areas with better social support provided mainly by social organizations, the empowering impact of the digital economy on older adult care services is more pronounced.

*Hypothesis 3*: The level of social support positively moderates the positive impact of the digital economy on older adult care services.

## 3 Research design

### 3.1 Sampling and data sources

This work examines panel data from 30 Chinese provinces (Tibet excluded due to extensive missing data) spanning 2013–2023 as the research sample. One of the reasons for choosing 2013–2023 is that the policies in this period gradually promote the integration of digitalization and older adult care services. The State Council released the “Several Opinions on Accelerating the Development of Elderly Care Services,” which first introduced the concept of informatization in older adult care services in 2013. In 2022, the “14th Five-Year Plan” for National Aging Development and Elderly Care Service System was unveiled, aiming to foster the deep integration of digitalization and older adult care services. Second, this stage shows the process of digital technology from a single point breakthrough to system integration enabling older adult care services. From mobile Internet to wearable devices to AI algorithm optimization to enhance service accuracy, digital technology has emerged as a crucial enabler for integrating “home-community-institution” older adult care services into a cohesive model. Third, in terms of data accessibility, China’s Ministry of Civil Affairs has been progressively releasing data on the count of older adult care institutions since 2013.

This study utilizes indicator data consistently measured across 2013–2023, employing linear interpolation and nearest-neighbor imputation methods to address minimal missing values, ultimately constructing an 11-year balanced panel dataset encompassing 30 provinces. The data of digital economy measurement, enterprise innovation input, control variables, older adult care service measurement (partly) and the number of social organizations come from the National Bureau of Statistics of China. The other part of older adult care service measurement data is from Statistics Yearbook regarding China Civil Affairs and Social. The number and output value of listed companies in the older adult care industry are from CSMAR.

### 3.2 Variable measurement and explanation

#### 3.2.1 Digital economy

Quantitative studies on digital economy (DE) have been abundant. First, a certain policy or a single dimension is taken as the proxy variable of DE. Second, the index system is constructed from the connotation of DE, which can be basically summarized as the arrangement and combination of “foundation,” “application,” “environment” and other elements. In this study, drawing on the research of Zhang et al. (51) and Liu et al. (52), the digital economy (DE) is categorized into three dimensions (see Table 1). The entropy method is utilized to conduct the calculation.

**Table 1:** Measurement index system of DE.

Dimensions	Indicators	Stats
Digital infrastructure	Number of internet broadband access ports	+
	Number of internet broadband access users	+
	Number of domain names per 1,000 people	+
	Mobile phone penetration	+
Digital industrialization	Total telecommunications business	+
	Software product revenue size	+
	Information services revenue scale	+
	Employment in urban units of information transmission, software and information technology services	+
	Number of legal persons in information transmission, software and information technology services	+
Industrial digitization	Number of computers used by enterprises per 100 people	+
	Proportion of e-commerce transactions among enterprises	+
	E-commerce transaction value	+
	Digital Financial Inclusion Index	+

#### 3.2.2 Older adult care services

Drawing on the research of Zuo et al. (53) and Xu and Zhang (54), this paper develops an indicator system encompassing three dimensions: “care service,” “medical service,” and “material help” (see Table 2 for details). They are, respectively, embodied in the care and love for older adults in all aspects of life, health-related services such as the provision of facilities for activities, and medical treatment and welfare subsidies for older adults. In this article, OACS is used as the abbreviation of older adult care services.

**Table 2:** Measurement index system of OACS.

Dimensions	Index	Stats
Care services	Number of nursing beds per 1,000 older adult people	+
	Number of nursing homes for older adults per 1,000	+
	Number of social workers (including assistant social workers) per 1,000 older adult persons	+
	Number of rehabilitation and medical outpatients in older adult care institutions	+
Medical services	Number of beds in medical and health institutions for older adults per 1,000	+
	Number of medical and health institutions for older adults per 1,000	+
	Number of health professionals per 1,000 older adult people	+
Material assistance	Number of facilities of community older adult care service institutions	+
	Construction area of community aged care service institutions	+
	Welfare expenditures for older adults	+
	Old-age benefits as a proportion of older adults	+

#### 3.2.3 Mediating and moderating variables

Innovation in older adult care-related corporations (CI) and the development of the older adult care industry (OACI) serve as the mediating variables in this paper. The National Bureau of Statistics has developed the Statistical Classification of the Elderly Care Industry (2020), which includes 12 categories such as education and training for older adults, human resources services, and financial services for older adults, based on the Classification of Industries of the National Economy (GB/T 4754–2017). This paper sorts out the national economic industry classification codes corresponding to these 12 categories, and information on listed companies belonging to the above industries are found in the CSMAR database and summarized according to their registration places and years. Then, the logarithm is taken. Based on the research of Hagedoorn and Cloodt (55), the R&D investment in older adult care-related corporations is used as the proxy variable of CI. The number of listed companies in the older adult care industry (OACI1) and the output value of older adult care industry listed companies (OACI2) are used to represent the development level of the older adult care industry at the provincial level. The moderating variable of this paper is the level of social support (SS) provided by social organizations. The number of social organizations (logarithm) is taken as the proxy variable for SS.

#### 3.2.4 Control variables

In line with existing relevant studies, variables such as regional socioeconomic status and aging conditions may exert an influence on older adult care services (56, 57). Thus, this work controls for the old-age dependency ratio (DR), measured as the ratio of the older adult population to the working-age population; the medical security level (MI), measured by the logarithm of the number of urban basic medical insurance participants; and the per capita disposable income (DI), measured by the logarithm of the per capita disposable income of all residents.

### 3.3 Model setting

Panel data regression is adopted. Equation 1 serves as the baseline regression model; Equations 2 and 3 are used for the mediation analysis; and Equation 4 is for the moderation analysis. The models are as following:


(1) $$\mathrm{OAC}S_{i,t}=\alpha_{0}+\alpha_{1}DE_{i,t}+\alpha_{2}\text{Control}s_{i,t}+\mu_{i}+\tau_{t}+\varepsilon_{i,t}$$

(2) $$\begin{array}{l} M_{i,t}(CI_{i,t}/\text{OACI}1_{i,t}/\text{OACI}2_{i,t})=\beta_{0}+\beta_{1}DE_{i,t}+\beta_{2}\text{Control}s_{i,t}+\\ \;\mu_{i}+\tau_{t}+\varepsilon_{i,t} \end{array}$$

(3) $$\mathrm{OAC}S_{i,t}=\delta_{0}+\delta_{1}DE_{i,t}+\delta_{2}M_{i,t}+\delta_{3}\text{Control}s_{i,t}+\mu_{i}+\tau_{t}+\varepsilon_{i,t}$$

(4) $$\begin{array}{l} \mathrm{OAC}S_{i,t}=\gamma_{0}+\gamma_{1}DE_{i,t}+\gamma_{2}SS_{i,t}+\gamma_{3}DE_{i,t}*SS_{i,t}+\\ \;\gamma_{4}\text{Control}s_{i,t}+\mu_{i}+\tau_{t}+\varepsilon_{i,t} \end{array}$$

### 3.4 Descriptive statistics

The descriptive statistics for the main variables are presented in Table 3. The average value of OACS is 0.264, with a standard deviation of 0.084. The minimum and maximum values are 0.040 and 0.456, respectively. This suggests that the overall level of older adult care services across provinces is moderate, and there are disparities among different regions. The average value of DE is 0.214, with a standard deviation of 0.128. The minimum value is 0.031, and the maximum value is 0.687. The distribution characteristics of the sample are largely in line with existing research on the digital economy, indicating that there are significant differences in the digital economy across provinces and that it remains in a relatively low overall state. The distribution characteristics of CI, OACI1, OACI2, and SS are rational and indicate that there are disparities in corporate technological innovation, older adult care industry development, and social support across different regions. The statistical distribution of other control variables is also reasonable.

**Table 3:** Descriptive statistics of main variables.

Variables	Obs	Mean	SD	Min	Max
OACS	330	0.264	0.084	0.040	0.456
DE	330	0.214	0.128	0.031	0.687
CI	330	21.359	4.272	0	26.094
OACI1	330	3.477	1.206	0.693	6.425
OACI2	330	19.670	1.581	14.710	23.134
SS	330	9.898	0.743	8.019	11.492
DI	330	10.178	0.398	9.301	11.348
DR	330	16.820	4.670	8.8	30.6
MI	330	7.846	0.881	5.200	9.330

## 4 Empirical analysis results

### 4.1 Baseline regression

Table 4 displays the inter-variable correlations within the baseline regression model. A significant positive correlation exists between DE and OACS at the 1% significance level, lending initial support to Hypothesis 1. The other control variables exhibited significant positive correlations with OACS, suggesting that the chosen control variables aligned with expectations. Moreover, to mitigate the potential impact of multicollinearity on the regression outcomes, a multicollinearity test is performed prior to the baseline regression. The results indicated that the mean Variance Inflation Factor (VIF) is 3.12, with all predictors having a VIF below 5, confirming the absence of multicollinearity issues.

**Table 4:** Results of correlation analysis.

Variables	OACS	DE	DI	DR	MI
OACS	1.000				
DE	0.577^***^	1.000			
DI	0.503^***^	0.832^***^	1.000		
DR	0.484^***^	0.459^***^	0.509^***^	1.000	
MI	0.463^***^	0.575^***^	0.362^***^	0.523^***^	1.000

The data in the table represent Pearson correlation coefficients. The symbols ***, **, and * denote significance at the 1, 5, and 10% levels, respectively.

Table 5 shows the baseline regression results of DE’s influence on OACS. According to model (1) and (2), the estimated coefficient of DE is significantly positive regardless of whether the control variable is added or not, which means that DE has a promoting effect on OACS. Models (3) and (4) show the regression results from 2013 to 2020 and 2021–2023, respectively. The former is the time period involved in most studies, and the latter is the time period of new data. 2021–2023 is the stage when the novel coronavirus epidemic forces the acceleration of digitization of older adult care services (such as contact-free services and online consultation), and it is also the stage where technology adaptability and penetration are significantly improved. The regression coefficients for DE in relation to OACS are robustly positive and statistically significant. Thus, Hypothesis 1 can be verified.

**Table 5:** The direct impact of DE on OACS.

Variables	(1) OACS	(2) OACS	(3) OACS	(4) OACS
DE	0.304^***^ (0.061)	0.272^***^ (0.062)	0.251^***^ (0.069)	0.526^**^ (0.236)
Control variables		√	√	√
Prov-fixed	√	√	√	√
Year- fixed	√	√	√	√
Observations	330	330	240	90
Adj R-squared	0.887	0.894	0.918	0.963

The table reports robust standard errors in parentheses. ***, **, and * indicate significance at the 1, 5, and 10% levels, respectively. This convention applies to all following tables.

### 4.2 Robustness test

The study employs a range of tests to ensure the robustness of the basic conclusions (see Table 6). First, to account for the lag effect of DE, the DE variable is shifted forward by 1 year and then regressed, which also helps to mitigate possible reverse causality. Subsequently, to mitigate the impact of outliers, all continuous variables are winsorized at the 1% level both upward and downward before being reintroduced into the regression model. To further control for the influence of other variables, GDP (logarithm) and the number of individuals aged 65 and over (logarithm) were added to the original set of control variables for regression analysis. Additionally, robust standard errors clustered at the level of the 30 provinces were utilized. The findings from models (1), (2), (3), and (4) closely align with the baseline regression results, thereby confirming the robustness of the conclusions.

**Table 6:** Robustness test results.

Variables	(1)	(2)	(3)	(4)	(5)	(6)
	OACS	OACS	OACS	OACS	DE	OACS
L. DE	0.310^***^ (0.077)					
DE		0.290^***^ (0.063)	0.301^***^ (0.065)	0.272^**^ (0.107)		0.471^***^ (0.093)
IV					0.002^***^ (0.000)	
Control variables	√	√	√	√	√	√
Prov-fixed	√	√	√	√	√	√
Year- fixed	√	√	√	√	√	√
Observations	300	330	330	330	330	330
Adj R-squared	0.905	0.895	0.894	0.894	–	–
Kleibergen-Paap rk LM statistic					32.84 [0.000]	
Kleibergen-Paap Wald rk F statistic					51.50 {16.38}	

Values within [] represent *p*-values, and values within {} indicate the critical values at the 10% significance level for the Stock-Yogo weak identification test. ***, **, and * indicate significance at the 1, 5, and 10% levels, respectively.

Simultaneously, to address potential endogeneity concerns, instrumental variables for DE were constructed and a 2SLS test was conducted. The number of fixed telephone users in 1984 serves as an indicator of the communication infrastructure development level in the region, which in turn influences the evolution and trajectory of DE to a certain extent. At the same time, there is no evidence to prove that the number of fixed telephone users in history is directly related to the older adult care service. Therefore, this instrumental variable fulfills the assumptions of relevance and exogeneity, rendering it a reasonable choice. Adapting the approach outlined by Nunn and Qian (58), the study constructs interaction terms between the 1984 fixed-line telephone user count per province and the prior-year internet user count, employing these as instrumental variables within the regression framework. In models (5) and (6), the coefficients associated with the instrumental variables in the first stage are found to be significantly positive. This finding suggests that the historical level of communication infrastructure has a positive effect on the development of the digital economy (DE). Moreover, these instrumental variables successfully pass the tests for relevance and strength, confirming their validity. Therefore, it is tested by instrumental variable method.

### 4.3 Mechanism analysis

The results of the mediation and moderation effect tests in this paper are presented in Table 7. As indicated by models (1) and (2), DE exerts a significant positive influence on CI. When DE and CI are included together in the regression model of OACS, the coefficients of both DE and CI remain significantly positive. This suggests that the digital economy can enhance the service level of regional older adult care by promoting innovation in older adult care-related corporations. Models (3), (4), (5), and (6) reveal that the regression coefficients of DE on both OACI1 and OACI2 are significantly positive. When OACI1 and OACI2 are individually incorporated into the regression alongside DE, the coefficients for DE, OACI1 and OACI2 all remain significantly positive. This finding suggests that the digital economy not only fosters the agglomeration of the regional older adult care industry but also boosts its output value, which in turn elevates the level of older adult care services. Thus, hypothesis 2 can be verified.

**Table 7:** Test results of mediating and moderating effects.

Variables	(1) CI	(2) OACS	(3) OACI1	(4) OACS	(5) OACI2	(6) OACS	(7) OACS
DE	1.293^*^ (0.756)	0.258^***^ (0.064)	2.430^***^ (0.270)	0.191^***^ (0.072)	2.887^***^ (0.548)	0.221^***^ (0.067)	0.047 (0.117)
CI		0.015^**^ (0.006)					
OACI1				0.033^**^ (0.013)			
OACI2						0.017^***^ (0.006)	
SS							0.062^***^ (0.015)
DE*SS							0.102^**^ (0.044)
Control variables	√	√	√	√	√	√	√
Prov-fixed	√	√	√	√	√	√	√
Year- fixed	√	√	√	√	√	√	√
Observations	330	330	330	330	330	330	330
Adj R-squared	0.996	0.895	0.990	0.895	0.974	0.896	0.900

***, **, and * indicate significance at the 1, 5, and 10% levels, respectively.

According to model (7), we can see that DE, SS and the product term DE*SS after their centralization are put into the model for regression, and DE*SS is significantly positive. The analysis reveals that the effect of the digital economy on older adult care services is significantly enhanced in regions with higher levels of social support, thus validating Hypothesis 3. Specifically, social support provided by social organizations may enhance the enabling role of the digital economy in older adult care services through several mechanisms. First, capacity building, which helps bridge the digital divide by addressing older adults’ difficulties in using technology. Social organizations can provide training to assist older adults in learning how to make online appointments, adapt to telemedicine services, and overcome obstacles in accessing and utilizing complex digital information (48, 49). Second, trust building, which alleviates the issue of low digital engagement caused by distrust of technology. Older adults often refrain from adopting digital tools due to perceived risks, distrust, and privacy concerns (59). Community-based organizations can offer interpersonal support and foster trust, thereby reducing anxiety and enhancing older adults’ willingness to participate in digital services (60, 61). Third, demand articulation, whereby social organizations collect the specific needs of older adult individuals and help promote the development of digital products better suited to their preferences (14, 62). Fourth, by leveraging digital platforms, social organizations can further integrate resources and respond to older adult needs with greater efficiency (72). Collectively, these mechanisms form a “last-mile” solution for effectively delivering digital technologies in the context of older adult care.

### 4.4 Regional heterogeneity analysis

Given the differences in resource endowments and stages of development, significant heterogeneity may exist between the more developed eastern regions and the less developed central and western regions. A comparative regional analysis is therefore conducted. Descriptive statistics indicate that DE is significantly higher in the eastern regions (*N* = 121, Mean = 0.304, SD = 0.150, Median = 0.271) than in the central and western regions (*N* = 209, Mean = 0.162, SD = 0.076, Median = 0.157). In contrast, OACS is only slightly higher in the eastern regions (*N* = 121, Mean = 0.279, SD = 0.099, Median = 0.288) compared to the central and western regions (*N* = 209, Mean = 0.256, SD = 0.073, Median = 0.267). As shown in Table 8, the heterogeneity analysis reveals that, regardless of whether control variables are included, the marginal effect of DE on OACS is significantly stronger in the central and western regions.

**Table 8:** Heterogeneity analysis: DE and OACS by region.

Variables	Eastern region		Central and western region	
	(1) OACS	(2) OACS	(3) OACS	(4) OACS
DE	0.229^***^ (0.087)	0.194^**^ (0.093)	0.437^***^ (0.120)	0.532^***^ (0.139)
Control variables		√		√
Prov-fixed	√	√	√	√
Year- fixed	√	√	√	√
Observations	121	121	209	209
Adj R-squared	0.866	0.873	0.899	0.911

***, **, and * indicate significance at the 1, 5, and 10% levels, respectively.

This finding may be attributed to the relatively well-established and saturated older adult care infrastructure in the eastern regions, where the benefits of rapid digital economy growth have already been largely realized. In such contexts, the role of DE is more about cost optimization rather than service expansion, functioning as a “nice-to-have” enhancement. By contrast, the central and western regions face more acute resource shortages and greater potential for development. In these areas, digital technologies can directly expand service accessibility, playing a critical “must-have” role and effectively filling service gaps. These findings highlight a stage-dependent pattern of digital older adult care development, wherein the marginal benefits of technology diminish as the overall development level increases. This also indirectly underscores the contribution of DE to narrowing regional disparities in older adult care services.

### 4.5 Further expansion: spatial spillover effect

Given the ability of the digital economy (DE) to efficiently transmit information across time and space, several empirical studies have explored the spatial spillover effects associated with informatization and the spatial correlation between aging-related undertakings (63, 64). This paper suggests that OACS in a given area is influenced by the local DE, as well as potentially affected by DE and OACS in neighboring regions. Hence, spatial measurement is carried out to test the possible spatial spillover effects. First, the spatial autocorrelation effects of DE and OACS for each year are assessed using Moran’s I index method, with the adjacency matrix serving as the basis for this analysis, as shown in Table 9. The results show that DE of each province has a significant spatial autocorrelation during 2013–2023, while the spatial autocorrelation of OACS is not significant. However, the existence of spatial autocorrelation in OACS and the incorporation of the spatial lag term of OACS into the model are not necessary conditions (65), so further spatial regression can be carried out and the results can be interpreted cautiously.

**Table 9:** Spatial “spread” characteristics of DE and OACS from 2013 to 2023.

Year	OACS		DE	
	Moran’s I	Z	Moran’s I	Z
2013	−0.169	−1.112	0.188^**^	1.923
2014	−0.179	−1.194	0.184^**^	1.916
2015	−0.168	−1.124	0.194^**^	1.993
2016	−0.179	−1.197	0.171^**^	1.780
2017	−0.129	−0.781	0.197^**^	1.980
2018	0.030	0.535	0.137^*^	1.482
2019	−0.023	0.100	0.170^**^	1.757
2020	0.071	0.881	0.157^**^	1.644
2021	0.076	0.921	0.163^**^	1.705
2022	0.097	1.097	0.160^**^	1.673
2023	0.103	1.147	0.166^**^	1.710

***, **, and * indicate significance at the 1, 5, and 10% levels, respectively.

Subsequently, a series of tests, including the LM test, LR test, Wald test, and Hausman test, are conducted under the adjacency matrix framework. These tests confirm that the SDM (Spatial Durbin Model) with spatio-temporal dual fixed effects is the most appropriate model. To further assess the robustness of the estimation, this paper also includes SEM (Spatial Error Model) and SAR (Spatial Autoregressive Model) with spatio-temporal double fixed effects for comparison. Table 10 shows that, to some extent, OACS are simultaneously influenced by both local DE and the DE and OACS levels in neighboring regions. Focusing on the results of SDM, both the spatial autoregressive coefficient of OACS and the spatial interaction term of DE are significantly positive. However, the regression coefficients of the spatial interaction terms cannot be directly interpreted as measures of spatial spillover effects, further effect decomposition is necessary (66). The decomposition results reported in the table show that the direct, indirect, and total effects of DE on OACS are all significantly positive. Specifically, the average direct effect of DE on OACS within a region is 0.238, while the indirect effect is 0.304. The total effect thus reaches 0.542. These findings suggest that the spillover effect of DE on OACS across neighboring regions is even stronger than its within-region impact.

**Table 10:** Regression results of spatial models of the impact of DE on OACS.

Variables	(1) SEM	(2) SAR	(3) SDM
DE	0.230^***^ (0.054)	0.236^***^ (0.053)	0.222^***^ (0.053)
lambda	0.253^***^ (0.078)		
rho		0.288^***^ (0.071)	0.238^***^ (0.076)
W* DE			0.186^**^ (0.084)
LR_Direct SZ	–	0.243^***^ (0.055)	0.238^***^ (0.055)
LR_Indirect SZ	–	0.093^***^ (0.036)	0.304^***^ (0.096)
LR_Total SZ	–	0.336^***^ (0.080)	0.542^***^ (0.118)
Control Variables	√	√	√
Prov-Fixed	√	√	√
Year- Fixed	√	√	√
Observations	330	330	330
LogL	747.104	749.804	752.263
R-sq within	0.371	0.370	0.379

Standard error in parentheses. ***, **, and * indicate significance at the 1, 5, and 10% levels, respectively.

A deeper analysis suggests that this positive spatial spillover effect may be realized through channels such as technology diffusion, resource flows, and learning-based coordination. First, the “core-periphery” diffusion mechanism of DE enables central cities—where talent, technology, knowledge, and capital are highly concentrated—to act as high-potential innovation hubs (67). Through digital platforms, these saturated resources can extend outward, enhancing the efficiency and quality of older adult care services in neighboring areas. Notably, DE has driven the digital transformation of the entire healthcare and older adult care ecosystem (68), helping to automate and digitize service delivery processes even in underdeveloped peripheral regions. This transformation ensures broader accessibility, equity, and availability of services (69), thereby narrowing interregional disparities in older adult care resources.

Second, spatial spillovers may arise from learning and synergy effects. The advanced practices of digital empowerment in pioneering regions often serve as models for replication through policy diffusion and emulation. At the same time, digital technologies foster collaborative governance by deeply integrating various stakeholders, enabling the formation of interregional systems for sharing healthcare and older adult care resources (70). Such systems optimize resource allocation and improve efficiency. For instance, technologies like telemedicine and big data analytics in health—combined with sensor-integrated mobile devices—allow older adults to access high-quality medical services across regions (27, 71). Therefore, DE’s advantages in integrating resources across space and time, accelerating information exchange, and facilitating multi-stakeholder collaboration play a critical role in promoting balanced development of older adult care services across regions.

## 5 Conclusions and implications

Combining the essential characteristics of DE and OACS, this paper clearly reveals the mechanism of DE’s influence on OACS. It is found that, first, DE has an obvious positive promoting effect on OACS. Second, DE improves OACS mainly by strengthening innovation in older adult care-related corporations and facilitating the growth of the older adult care industry. Third, the enhancing effect of DE on OACS is positively moderated by the social support provided by social organizations, that is, the better the social support, the more obvious the enhancing effect of DE on OACS. Fourth, to a certain extent, DE can also affect the development of OACS in neighboring provinces.

The findings of this paper significantly advance the existing research on the digital economy (DE) and older adult care services, offering valuable theoretical insights for a deeper exploration of the intricate interplay and influence mechanisms among the digital economy, social governance, and older adult care services. First of all, the paper verifies that the technological innovation and industrial transition driven by the digital economy can further spill over to the field of social services, including older adult care. Second, this study merges scholarly concerns regarding the digital economy and social governance, demonstrating that the synergistic development of the digital economy and social organizations can significantly bolster the improvement of older adult care services. Third, the study further demonstrates that the digital economy can generate spatial spillover effects not only in economic development but also in social services, including older adult care services.

The findings of this paper also hold significant practical implications, offering valuable policy insights and bolstering confidence in enhancing older adult care services through the development of the digital economy and the increased involvement of social organizations. First, we should prioritize the digital economy as the catalyst to drive industrial innovation, foster the agglomeration and development of the older adult care industry, and enhance the integration of the innovation chain and the industrial chain, thereby elevating the quality of older adult care services. At the same time, development goals should be differentiated based on regional endowments. In regions with relatively strong foundations in digital infrastructure and older adult care services, efforts should focus on breaking through the bottleneck of “digital involution” by integrating cutting-edge technologies such as artificial intelligence, and shifting from mere technological application toward institutional innovation to cultivate new competitive advantages. In contrast, less developed regions should accelerate the construction of digital infrastructure, enhance the efficiency of converting digital investment into service provision, and leverage digital tools to connect with the rich healthcare and older adult care resources in more advanced regions. Second, social organizations should be promoted to participate in community social governance, exert their ability to leverage and link the resources of governments, enterprises, families and other parties, so that they can handle the needs of older adults with professional knowledge, voluntary spirit and higher efficiency, provide emotional and other support, and assist older adults in adapting to the digital society. Third, leverage the spatial diffusion and spillover effects of the digital economy to foster balanced development of regional older adult care services.

This study is subject to several limitations. First, the research employs panel data at the provincial level in China. However, this dataset fails to account for the significant heterogeneity among cities within provinces. Consequently, it cannot fully eliminate the potential confounding effects of intra-provincial disparities on the empirical results. Second, this paper focuses solely on the moderating effect of social organizational support on the impact of the digital economy on older adult care services, without examining the potential contributions of other support systems such as family and government support. Future research can first collect relevant data at the city level to check whether there are differences in conclusions. In addition, it can broaden the impact of multi-subject support or governance, including government and enterprise support, on older adult care services in the context of the digital economy.

## Data Availability

The original contributions presented in the study are included in the article/supplementary material, further inquiries can be directed to the corresponding authors.
